# Genetic Diversity of the Hepatitis B Virus Strains in Cuba: Absence of West-African Genotypes despite the Transatlantic Slave Trade

**DOI:** 10.1371/journal.pone.0125052

**Published:** 2015-05-15

**Authors:** Licel A. Rodríguez Lay, Marité B. Corredor, Maria C. Villalba, Susel S. Frómeta, Meilin S. Wong, Lidunka Valdes, Marcia Samada, Aurélie Sausy, Judith M. Hübschen, Claude P. Muller

**Affiliations:** 1 Pedro Kourí Institute of Tropical Medicine, Havana, Cuba; 2 Centro de Investigaciones Médico-Quirúrgicas, CIMEQ, Havana, Cuba; 3 Laboratory of Immunology, Luxembourg Institute of Health, Esch-Sur- Alzette, Grand-Duchy of Luxembourg; Centers for Disease Control and Prevention, UNITED STATES

## Abstract

Cuba is an HBsAg low-prevalence country with a high coverage of anti-hepatitis B vaccine. Its population is essentially the result of the population mix of Spanish descendants and former African slaves. Information about genetic characteristics of hepatitis B virus (HBV) strains circulating in the country is scarce. The HBV genotypes/subgenotypes, serotypes, mixed infections, and S gene mutations of 172 Cuban HBsAg and HBV-DNA positive patients were determined by direct sequencing and phylogenetic analysis. Phylogenetic analysis of HBV S gene sequences showed a predominance of genotype A (92.4%), subgenotype A2 (84.9%) and A1 (7.6%). Genotype D (7.0%) and subgenotype C1 (0.6%) were also detected but typical (sub)genotypes of contemporary West-Africa (E, A3) were conspicuously absent. All genotype A, D, and C strains exhibited sequence characteristics of the adw2, ayw2, and adrq serotypes, respectively. Thirty-three (19.1%) patients showed single, double, or multiple point mutations inside the Major Hydrophilic domain associated with vaccine escape; eighteen (10.5%) patients had mutations in the T-cell epitope (amino acids 28-51), and there were another 111 point mutations downstream of the S gene. One patient had an HBV A1/A2 mixed infection. This first genetic study of Cuban HBV viruses revealed only strains that were interspersed with strains from particularly Europe, America, and Asia. The absence of genotype E supports previous hypotheses about an only recent introduction of this genotype into the general population in Africa. The presence of well-known vaccine escape (3.5%) and viral resistance mutants (2.9%) warrants strain surveillance to guide vaccination and treatment strategies.

## Introduction

Despite advances in the prevention, diagnosis and treatment of hepatitis B, this viral infection continues to be a major public health concern in many countries. The human hepatitis B virus (HBV) is the prototype of the *Hepadnaviridae* family with a partially double-stranded circular DNA genome of about 3.200 nucleotides organized into four open reading frames [1]. Since its polymerase lacks proof-reading capacity, HBV has developed into at least eight main genotypes, namely genotypes A to H, which differ from each other by at least 8% in the complete nucleotide sequence, and into a number of subgenotypes [2]. Recently additional genotypes I and J have been proposed [3, 4]. All these genotypes exhibit distinct geographic distributions. Genotypes A and D co-exist in many areas of the world, such as Western Europe, America, India and parts of Africa, while genotype C predominates in China, Japan, and Southeast Asia [5]. Genotype A comprises potentially seven subgenotypes (A1 to A7), all of which are found in Africa. Subgenotype A1 is found throughout East-Africa and A3 to A7 mainly in West- Africa [6]. A2 occurs only sporadically in Africa. Genotype D is also divided into several subgenotypes and is the most prevalent and the most widely distributed HBV genotype in Western populations and in other parts of the world including Northern Africa [5, 7]. In contrast, genotype E is highly prevalent throughout West-Africa but essentially restricted to the sub-Saharan genotype E crescent expanding from Senegal to the Central African Republic and to Namibia in the South [8]. The high prevalence and the extensive spread within Africa are in sharp contradiction with the very low genetic diversity of genotype E strains. This has led to speculations that this genotype E has only been recently introduced in the general African population [9].

It has been suggested that some genotypes are associated with a more severe clinical course and a weaker response to antiviral treatments. Therefore, genotype information is also of interest for the management of chronic hepatitis B [2].

The HBV surface antigen (HBsAg) is the major envelope lipoprotein. The major antigenic or 'a' determinant of this surface protein (amino acids (aa) 124 to 147) induces virus-neutralizing antibodies and is the target of protective antibodies induced by HBV vaccines. Depending on their ‘a’ determinant, HBV strains are grouped into different serotypes which show some preferential associations with certain genotypes [10]. Within the Major Hydrophilic domain (MHD, aa 99–169) the G145R substitution is the most common mutation associated with vaccine-escape [11]. Some other important escape mutations have been observed in the HBV genome [1].

Cuba is an HBsAg low prevalence country, and there is very little information about circulating HBV genotypes/subgenotypes and serotypes. Since 1992 hepatitis B vaccine is included in the National Immunization Program and all newborn children are vaccinated at birth. The vaccine is also offered to the previously identified risk groups for HBV infections. The Cuban population is essentially the result of a population mix of Spanish descendants and former African slaves who were deported to Cuba until the second half of the 19^th^ century. The present study investigated the genetic diversity of the HBV strains in Cuba to provide guidance for public health programs and to potentially learn which genotypes may have been present in Africa centuries ago during the slave trade. The typical African genotype E strains were conspicuously absent in Cuba, supporting previous hypotheses of an introduction of this genotype only after the slave trade came to an end.

## Materials and Methods

### Ethics statement

Serum samples were collected by trained staff at the main Gastroenterology Medical practice in Cuba and in hospitals or outpatient clinics for molecular diagnostics of hepatitis B in the frame of the Viral Hepatitis National Surveillance Programme. Taking a blood sample for diagnostics requires only verbal informed consent according to Cuban legislation and this consent was obtained from all patients or their guardians and was recorded in the medical records of the patients by the physicians in charge. Directly after receipt of the sample by the National Reference Laboratory for Viral Hepatitis, a unique identifier was attributed and subsequently used for all analyses. Only three investigators had access to both the patients’ names and the unique identifier. These investigators collected patient data from the laboratory request sheets completed by the treating physicians or from additional information sent together with the request or enquiry sheets by the epidemiologists in charge and added this information to the anonymized sample records prior to analysis. In case the exact age was unknown, patients were attributed to the children age group (0–18 years) if samples came from pediatric hospitals and to the adult age group (19 years and older) if the samples came from hospitals for adults. The study was approved by the Ethics Committee of the Pedro Kouri Institute of Tropical Medicine in compliance with the Declaration of Helsinki (number CEI-IPK 11–12).

### Study population and sample collection

Two-hundred serum samples of 172 HBsAg and HBV-DNA positive patients were received for HBV molecular diagnosis between 2006 and 2011. From twenty patients two or more samples were available. In these cases the first sample was used for genotype and serotype analysis. Subsequent samples were only included in the analysis whenever there were discrepancies to the first sample, namely mutations or mixed infections. Samples were stored at—20°C until used by the National Reference Laboratory on Viral Hepatitis at the Pedro Kouri Institute of Tropical Medicine in Havana.

Demographical, epidemiological and clinical characteristics of the study population are summarized in Table 1. Essentially all adults were born before the introduction of routine childhood HBV vaccination.

**Table 1:** Demographics, epidemiological and clinical characteristics of the 172 HBV-infected patients included in the study.

*Parameters*	*N*	*%*
**Gender**		
Male	66	38.4
Female	31	18.0
Unknown	75	43.6
**Age**		
Children (0–18 years)	20	11.6
Adults (≥19 years)	152	88.4
**Probable route of infection**	**Vertical: 9**	5.2
	**Sexual: 27**	15.7
	**Parenteral: 31**	18.0
	**Unknown: 4**	2.3
	**No data available: 101**	58.8
Clinical status	Acute infection: 3	1.7
	*De novo* infection after liver transplantation: 2	1.2
	Chronic infection: 167	97.1
Co-infections	HBV/HCV: 7	4.06
	HBV/HIV: 21	12.2

### DNA extraction, PCR amplification and phylogenetic analysis

DNA was extracted from 200 μL of serum using the QIAamp DNA mini kit (Qiagen GmbH, Hilden, Germany) according to the manufacturer’s instructions. The HBV S gene was amplified and sequenced as previously described [12]. Sequences were edited with SeqScape v2.5 (Applied Biosystems, Foster City, CA) and aligned with reference sequences and with sequences obtained by BLAST using BioEdit Sequence Alignment Editor (version 7.0.9.0, Ibis Biosciences, Carlsbad, CA). Samples with more than 8 ambiguous nucleotides in the S fragment sequence were cloned using the pCR4-TOPO kit (Invitrogen, Merelbeke, Belgium) to see whether the patients were infected with different (sub-) genotypes.

jModelTest (http://darwin.uvigo.es/our-software) using the “Akaike information criterion” selection strategy was applied to choose the appropriate model of nucleotide substitution. Based on these results, phylogenetic analyses were conducted with MEGA version 6 (www.megasoftware.net) using the maximum likelihood method and the General Time Reversible model (GTR) with gamma distributed rates with invariant sites (G+I). As a measure of the robustness of each node, the bootstrap method (100 pseudo-replicas) was used.

Sequences of the Cuban HBV strains characterized in the present study have been submitted to EMBL and GenBank Nucleotide Sequence Databases under the following accession numbers: HE981728, HE981729, KP144200—KP144210 and KP165620—KP165837.

### Prediction of serotypes and detection of mutations

The nucleotide sequences obtained from the S gene were also translated into the corresponding aa using the BioEdit Sequence Alignment Editor. The serotype was predicted from the aa at positions 122 (Lys/Arg for *d/y* determinants), 160 (Lys/Arg for *w/r*), 127 (Pro/Thr/Leu-Ile for *w1-2/w3/w4*), 177 (Val for *q+*; Ala for *adrq-*), and 178 (Pro for *q+*; Gln for *adwq-*). Discrimination between *ayw1* and *ayw2* was based on positions 134 and 159 (Phe or Ala, for *ayw1* and Tyr or Gly, for *ayw2*) [10, 13].

## Results

### HBV genotypes and phylogenetic analysis

**Fig 1:**
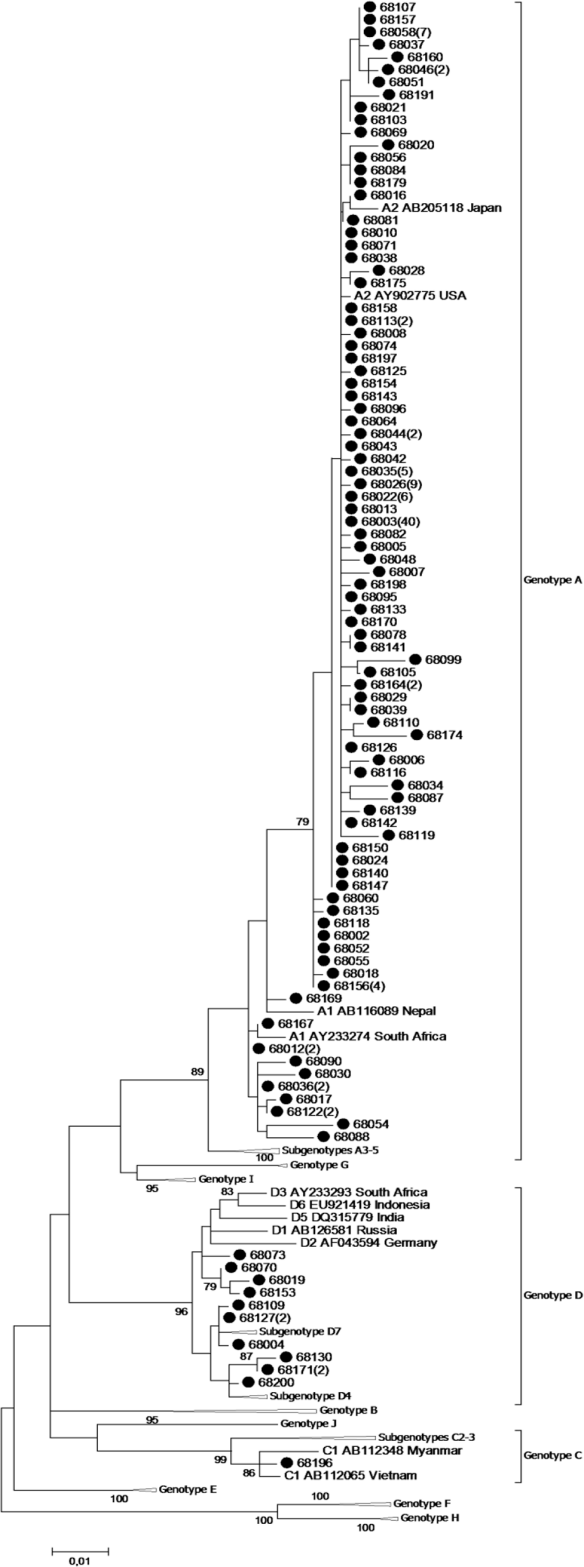
Phylogenetic tree based on the HBV S gene sequences of all strains detected in Cuba. Phylogenetic tree based on the maximum likelihood method and the General Time Reversible model with gamma distributed rates with invariant sites of the HBV S gene sequences of all strains detected in Cuba.

**Fig 2:**
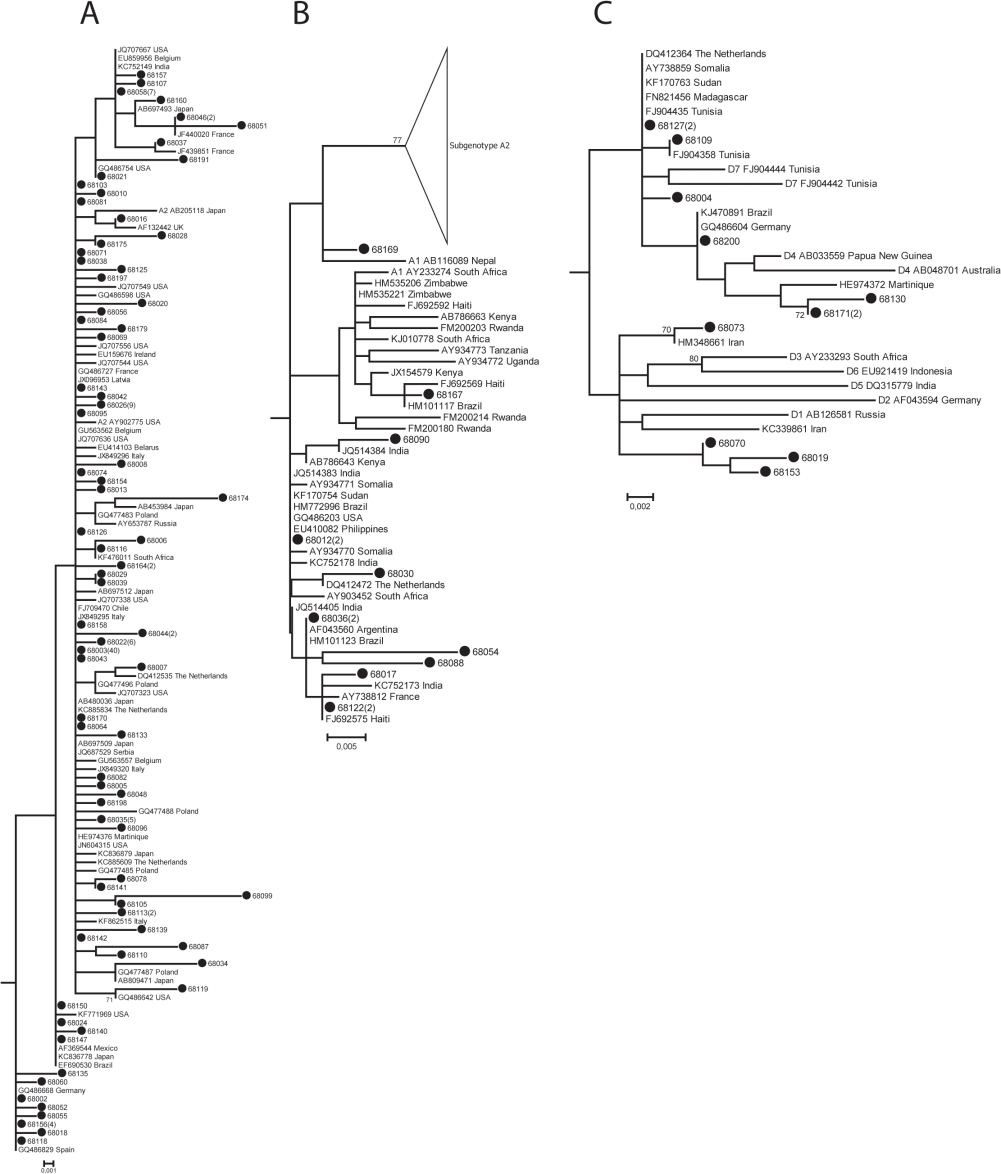
Phylogenetic comparison of the Cuban strains with sequences downloaded from GenBank. Phylogenetic comparison of the strains of Fig 1 to identical or similar strains identified by BLAST and other strains from Africa and Haiti downloaded from GenBank. a) Subgenotype A2 cluster. b) Subgenotype A1 cluster. c) Genotype D cluster.

Phylogenetic analysis of 681 nucleotides of the HBV S gene showed that the sequences from Cuba clustered with reference sequences of genotypes A, C and D. Most HBV strains belonged to genotype A (159/172, 92.4%), subgenotypes A2 (84.9%) and A1 (7.6%) (Fig 1). Among the A2 sequences there was one major variant (68003) detected in 40 patients (Fig 2A), while for A1 a maximum of 2 patients had identical S gene sequences (Fig 2B). Genotype D was the second most common genotype (12/172, 7.0%) and besides 4 samples each of subgenotypes D4 and D7, there were another 4 sequences for which the subgenotype could not be unequivocally determined based on the S gene sequence alone (Figs 1 and 2C). Only one strain belonged to subgenotype C1 (0.6%, Fig 1). Four samples were suspected to have mixed infections. One A1/A2 mixed infection of a patient who one year earlier was already infected by HBV subgenotype A2 (68085) was confirmed by cloning.

The Cuban strains are marked by black dots and the numbers in brackets indicate how often that variant was found. Each reference strain is identified by genotype and subgenotype, Gen-Bank accession number and country of origin. Numbers at the nodes indicate bootstrap percentages over 100 replicates (only values ≥ 70 are shown). The bar indicates genetic distance.

All patients co-infected with HIV (n = 21) were infected with genotype A, mostly with subgenotype A2 (19/21, 90.5%). Also all patients co-infected with HCV (n = 7) and with acute hepatitis B (n = 3) were infected with subgenotype A2. Of 9 infants born to HBsAg positive mothers, 7 (77.7%) were infected with subgenotype A2 and two (22.2%) with genotype D.

The Cuban genotype A2 strains clustered interspersed with strains mainly from throughout Europe, the US and Japan (Fig 2A). Cuban A1 strains were most similar to viruses from North and South America, Haiti, Asia and to a lesser extent Europe. With a few exceptions, they showed little resemblance to contemporary African A1 sequences. HBV genotype D strains from Cuba grouped with strains reported from Europe, South America, Asia and North and East Africa (Fig 2C).

### Serotypes

According to the S gene sequences we found serotypes a*dw2*, *adrq and ayw2* in the samples analyzed. All of the *adw2* serotype strains were of genotype A (n = 155, 90.1%), with the majority of them grouping with subgenotype A2 (93.6%). Moreover, all ayw2 strains belonged to genotype D (n = 12, 7.0%), and the single adrq strain belonged to the single C1 strain (0.6%). For four strains (2.3%) of genotype A (3 and 1 from subgenotype A1 and A2, respectively) a serotype cannot be attributed due to mutations in aa 122, 127 and/or 134.

### MHD mutations of the HBs protein and other envelope mutations

The presence of aa mutations potentially involved in vaccine escape, diagnostic failure, failure of hyperimmunoglobulin (HBIG) treatment, or resistance to antiviral therapy was analyzed. Overall there were 33 (33/172, 19.1%) patients with single, double or multiple point mutations inside the MHD associated with immune escape (Table 2). These mutations were found in 6 (6/13, 46.1%) patients of subgenotype A1, in 26 (26/146, 17.8%) patients of subgenotype A2 and in the patient infected with subgenotype C1. Mutations in the B-cell epitope of the S gene (aa 124–147) were found in 6 (6/172, 3.5%) patients (Table 2). The most frequent mutations were Y161F (13/33, 39.3%) and E164D (11/33, 33.3%). The most common vaccine-escape mutation G145R was present in a patient infected by the parenteral route and living in the eastern region of the country. Mutation Y161F was found in patients with *de novo* HBV infection after liver transplantation, in a heterosexual couple infected through sexual route and in 3 patients infected by parenteral route. None of 9 infants born to HBsAg positive mothers exhibited vaccine-escape mutations. Four of them received hepatitis B vaccine at birth, and 5 received also HBIG. Double mutation E164D/I195M was found in 5 (2.9%) patients, four of them (4/21, 19%) were men who have sex with men and co-infected with HIV.

**Table 2:** Prevalence of mutations associated with immune escape within the S gene, including a B cell epitope (amino acids 124–147, bold) and other MHD mutations.

Subgenotype/Serotype	MHD Mutations	n	%
A1/adw2	I110M, **M133T, F134I**	1	3
	K122R, **F134L**, **D144E, G145R**	1	3
	**F134L**	1	3
	Y161F	1	3
	E164D	1	3
	E164D/I195M	1	3
A2/adw2	Y161F	11	33.3
	E164D	4	12.1
	M103I, S154P	1	3
	I110L	1	3
	T114N	2	6.1
	K122E, T123A, **P127A**	1	3
	**G130N**	1	3
	E164D/I195M	4	12.1
	Y161F, E164D	1	3
C1/adrq	W104, I117S, **T126I**	1	3
**Total**		**33**	**100**

Eighteen patients (18/172, 10.5%) had mutations in a major T-cell epitope (aa 28–51). Mutations were found in 2 (2/13, 15.4%) patients of subgenotype A1 and in 16 (16/146, 10.9%) patients of subgenotype A2 (Table 3). The most frequent mutation was S45A/P (13/18, 72.2%). Several strains showed mutations downstream of the MHD region (111 point mutations), including V194A (n = 11, 10%), I195M (n = 15, 14%), and S210R (n = 14, 12.7%).

**Table 3:** Prevalence of mutations associated with the T-cell epitope (aa 28–51) within the S gene.

Subgenotype/Serotype	T-cell epitope mutations	n	%
A1/adw2	W36L, N40S, L49H	1	5.5
	S45P, L49R, Q51P	1	5.5
A2/adw2	N40S	2	11.1
	G44E, S45A	1	5.5
	S45A	9	50
	S45P	2	11.1
	P46T	1	5.5
	L49R	1	5.5
	**Total**	**18**	**100**

## Discussion

Analyzing 172 HBV strains from Cuba we found more than 92% genotype A sequences, most of which were of the A2 subgenotype, and 7% genotype D sequences. Thus, a clinically more favorable genotype dominates [2, 14]. Most of the strains were A/*adw2* (90.1%) and D/*ayw2* (7.0%).

The Cuban population is essentially the result of a population mix of Spanish descendants and former African slaves. The Africans originated mostly from the Gulf of Guinea and the South of Angola, with a minor proportion from Central Africa and exceptionally from the East Coast [15–17]. Amerindian tribes that originally inhabited the island and Asiatic descendants play only a minor role [15]. The vast majority (>80%) of the Cuban sequences belonged to the European-North American group of subgenotype A2. They did not form a separate cluster, but were interspersed with contemporary strains suggesting multiple recent importations rather than introductions by early colonial settlers. While A2 strains are essentially absent from sub-Saharan Africa, A1 strains are dominant in contemporary East Africa [18]. The few Cuban A1 strains did not form a distinct cluster, but again were interspersed with contemporary sequences from around the world and not preferentially with East African A1 strains. Only a small population of slaves deported to Cuba originated from East Africa and there is no phylogenetic indication that they introduced the Cuban A1 strains. Thus both A1 and A2 strains seem to be recent introductions from across the world. The genotype A3 strains typical for the Bight of Benin, the former Gold Coast and todays Nigeria were not found in Cuba. Even more surprisingly genotype E was conspicuously absent in Cuba. This genotype is highly endemic throughout the “genotype E crescent” spanning from Senegal in the West to Central African Republic in the East and Namibia in the South [8]. This vast region includes most of the territories where the slaves were captured before their force-migration to the Caribbean. The absence of genotype E from Cuba is in line with our previous finding of a low prevalence of genotype E strains in Haiti that were interspersed with current genotype E strains from Africa as an indication of only recent introductions [19]. In Haiti, where most of the population descends directly from African slaves, we also found the potential subgenotype A5 previously reported from Nigeria [19] and later from Cameroon [6]. We concluded that genotype E was absent when and where the African slaves were rounded up and that the potential subgenotype A5 predated the introduction or spread of genotype E throughout West-Africa [19]. The failure to find the supposedly early West-African A5 strains in Cuba may be due to their very limited prevalence along the Gulf of Guinea.

Genotype D was the second most prevalent genotype found in our study, but the subgenotypes could not be determined in all cases on the basis of the S gene sequence alone. This genotype was found in three children and eight unrelated adults living in different parts of the country. At least one of the adults had been infected abroad. Also this genotype seems to be a contemporary introduction into Cuba.

Subgenotype C1 is normally of Asian origin. Today only 1% of the Cuban population is of Asian origin [15]. Although rare in Cuba, this genotype correlates with high risk of cirrhosis, hepatocellular carcinoma and poor prognosis [20].

Genotypes F and H were not found in Cuba although they are indigenous to Latin American natives with prevalences of genotype F up to more than 80% in Colombia and Venezuela [21]. Historical and anthropological accounts report that the native population was essentially eradicated by 1550, few years after the Spanish conquest. Small communities of natives survive in the Eastern parts of the island [16]. The present study included samples from the five Eastern provinces but genotype F was not detected, similar to our results in Haiti [19]. Thus in contrast to Haiti where the African A5 strains were found, no strains indigenous to America or West Africa from where most of the slaves originated were detected. We cannot totally exclude that conditions in Cuba were not conducive to vertical transmission and that, despite the long sample collection throughout the country, descendants other than of African slaves may be overrepresented, but otherwise our results are highly suggestive that in particular genotype E, highly prevalent and ubiquitous throughout West-Africa, was only introduced after the slave trade came to an end.

Genetic variability in the HBsAg can undermine vaccine efficacy [1, 22]. Mutations inside the MHD were less frequent (19.1%) than in European countries including Spain (39%) and France (27.8%) [23, 24]. The G145R mutation was associated with the typical changes in residues 122, 134 and 144 of the MHD. The double mutation D144E/G145R can potentially restore the replication capacity in lamivudine-resistant strains. This double mutation does not only confer resistance against antivirals but more importantly also against the vaccine [22]. We detected the Y161F mutation in the two patients with *de novo* HBV infection after liver transplantation similar to previous reports [25]. Although vaccine escape mutations seem to be less prevalent in Cuba, thorough molecular surveillance is warranted.

Other mutants within the MHD may affect the sensitivity of HBsAg detection assays [26, 27], such as T126I which has been reported so far only in genotype B and C [28] including in our single C1 sample. The double S protein mutation E164D/I195M found in 5 samples is generated by the triple polymerase mutation rtV173L/L180M/M204V and is associated with antiviral resistance. The E164D/I195M mutation is a consequence of the pol/S gene overlap and is of particular public health relevance because most antiviral resistant HBV strains also overcome vaccine-induced immunity infecting even immunized individuals [29]. The increasing use of antiretroviral drugs in HIV/HBV infected, immunosuppressed patients also in Cuba facilitates the emergence of new HBV mutants [30]. In 2010, 5.692 HIV seropositive patients were under antiretroviral treatment in Cuba [31]. Thus clinical case management needs to be optimized, HIV-infected patients should be vaccinated against HBV and surveillance for drug-resistant viruses should be strengthened.

## Conclusions

This first molecular study of Cuban HBV strains provides guidance for patients’ management and for the surveillance of antiviral resistant and vaccine-escape mutants. We detected almost exclusively genotypes A and D that were probably all introduced relatively recently into the country. The conspicuous absence of the typical Western African genotype E and A3 strains suggests that these were rare when and where the slaves were rounded up in Africa. This supports our previous hypothesis of a relatively recent spread of these genotypes in the general African population.
